# Delayed discharge from post-anesthesia care unit: A 20-case retrospective series

**DOI:** 10.1097/MD.0000000000035447

**Published:** 2023-10-27

**Authors:** Xiaohui Liu, Yimin Zhang, Xingzhi Cai, Huili Kan, Ailan Yu

**Affiliations:** a Department of Anesthesiology, Liaocheng People’s Hospital, Liaocheng, Shandong, China.

**Keywords:** delayed discharge, post-anesthesia care unit, postoperative recovery period

## Abstract

**Objective:**

To summarize the characteristics of patients with delayed discharge from the post-anesthesia care unit and to analyze the factors and outcomes of delayed discharge.

**Methods:**

Twenty cases of delayed discharge from the PACU (PACU stay >2 hours after surgery) of the main operating room in Liaocheng People’s Hospital, a class A tertiary comprehensive hospital, between January 1, 2021, and December 31, 2022, among 28,084 patients who were transferred to the PACU from the operating rooms after surgery, were retrospectively analyzed. The collected data included patient characteristics, American society of anesthesiologists grade, information related to surgery and anesthesia, and outcomes. The factors for delay were assigned to 1 of 6 groups: delayed recovery from anesthesia, surgical complications, cardiovascular instability, hypoxia, inadequate analgesia, and waiting for the operating room.

**Results:**

The incidence of delayed discharge from PACU was 0.7‰. Among 20 patients, more than half of the patients were over 65 years of age, American society of anesthesiologists grade II~III, body mass index <30 kg/m^2^, and urological surgery (7, 35%), liver surgery (4, 20%), thoracic surgery (4, 20%) accounted for a relatively high proportion. Nineteen (95%) patients received general anesthesia with or without peripheral nerve block. The main factors included delayed recovery from anesthesia (6, 30%), surgical complications (5, 25%), cardiovascular complications (4, 20%), hypoxia (3,15%). After discharge from the PACU, 1 (5%) died in the intensive care unit, and the other 19 (95%) patients were safely discharged from the hospital.

**Conclusion:**

The incidence of delayed discharge from the PACU was low, and it was more likely to occur in the elderly, during major operations, and under general anesthesia. Delayed recovery from anesthesia was the most common factor. Most patients were safely discharged from the hospital.

## 1. Introduction

The post-anesthesia care unit (PACU) is a special area that provides centralized observation and care for postoperative patients with the aim of reducing postoperative morbidity and mortality.^[1,2]^ PACU was an important transition between intraoperative and postoperative monitoring of patients, and an important place to improve work turnover efficiency.^[3]^ In the PACU, patients are characterized by rapid turnover and changes in their condition. During the early stage of the patient recovery of physiological function from anesthesia and operation, intensive monitoring, care, and necessary treatment were provided by anesthesiologists and anesthesia nurses until they could be safely transferred to a special area.^[4]^

The length of stay in the PACU was determined based on postoperative recovery. According to expert opinions from the Chinese Association of Anesthesiologists, if the duration of stay in the PACU was >2 hours, it was considered a delayed discharge from the PACU. The rate of delayed discharge from the PACU after anesthesia is a quality control indicator of anesthesia in China, reflecting the level of operation, anesthesia management, and quality of anesthesia in medical institutions. Prolonged stay in the PACU not only increases the cost to patients but also adversely affects patient and surgeon satisfaction.^[5]^ At the same time, it reduces the turnover rate of PACU beds, leading to an increase in operating costs.

This article reviewed cases of delayed discharge from the PACU after surgery in recent years at a Class A tertiary comprehensive hospital in China, analyzed the current situation, and provided a clinical reference for further improving the recovery quality of anesthesia patients, the level of surgical anesthesia management, and bed utilization rate and work efficiency.

## 2. Methods

### 2.1. Study design and setting

This retrospective study was conducted at Liaocheng People’s Hospital between January 1, 2021, and December 31, 2022. Owing to the retrospective and anonymous nature of this study, the Medical Ethics Committee of Liaocheng People’s Hospital provided written informed consent.

Liaocheng People’s Hospital is a 3200 bed public Class A tertiary comprehensive hospital with 4 campuses. The main operating room suite had 19 operating rooms and an 8 bedded PACU. Approximately 18,000 patients pass through the PACU after surgery each year, and 6000 patients are sent directly to ward, cardiac, or general intensive care units (ICU). Patients who were not admitted to the PACU were excluded. Patients undergoing neurosurgical procedures; obstetrics and gynecology procedures; eye, ear, nose, and throat procedures; and plastic surgical procedures were also excluded because these procedures were not performed in the main operating room.

Our PACU was staffed from 8 am to 22 pm Monday through Saturday. The anesthesia nurse-to-patient ratio varied from 1:1 to 1:3, depending on the patient condition and staffing. An attending anesthesiologist was on duty in the PACU and responsible for deciding whether to transfer patients out of the PACU. In general, surgical patients have their own beds in the ward before surgery, except for outpatient emergency surgery, and they prefer to return to their respective wards after surgery. Patients admitted to the PACU included those who: receiving general anesthesia and tracheal tube or laryngeal mask removed after non-cardiac surgery, if available; receiving spinal or epidural anesthesia; receiving nerve block or local anesthesia combined with intravenous analgesic and sedative drugs, and could not meet the criteria of returning directly to the ward after surgery. Heart rate, blood pressure, oxygen saturation, and body temperature were monitored routinely in the PACU. The degree of pain and complications related to surgery and anesthesia were routinely evaluated and treated when necessary. The criteria for patients discharged from the PACU to the general ward included: fully conscious; airway patency, airway protective recovery, respiration, and oxygenation recovery to the preoperative baseline level; circulatory stability, no unexplained arrhythmia or severe bleeding, and cardiac output ensuring adequate peripheral perfusion; pain and postoperative nausea and vomiting were controlled; temperature within the normal range; and no surgical and anesthesia-related complications that required management in the PACU. Patients in an unstable condition were transferred to the ICU if they could not be safely transferred back to the ward. PACU duration was defined as the time from admission to discharge from PACU. Delayed discharge from the PACU was defined as a stay in the PACU for >2 hours, according to the quality control indicators of anesthesia in China.

### 2.2. Study population

We analyzed all cases of delayed discharge from the PACU of the main operating room in Liaocheng People’s Hospital between January 1, 2021, and December 31, 2022, in 28,084 patients who were transferred to the PACU from operating rooms after surgery. Twenty patients who met the criteria for delayed discharge were included in this analysis.

### 2.3. Data collection procedure and analysis

Data including age, sex, body mass index (BMI), American society of anesthesiologists (ASA) grade, procedure type, procedure duration, anesthesia duration, and PACU stay duration were extracted from the Do-Care anesthesia system. The quality management team of the department of anesthesiology, composed of 3 consultant anesthesiologists and 2 senior anesthetic nurses, analyzed the causes of delayed discharge from the PACU according to the patient medical records. The factors for delay were assigned to 1 of 6 groups: delayed recovery from anesthesia, surgical complications, cardiovascular instability, hypoxia, inadequate analgesia, and waiting for the operating room (Table 1).

**Table 1:** Factors for delayed discharge from PACU.

Group
1. Delayed recovery from general anesthesia
2. Surgical complications
3. Cardiovascular unstable
4. Hypoxia
5. Inadequate analgesia
6. Waiting for operating room

PACU = post-anesthesia care unit.

Data was analyzed by descriptive analysis, continuous variables were expressed as the mean ± standard deviation if they were normally distributed or described as median (interquartile range) if they were non-normally distributed. Categorical variables are presented as counts (percentages, %).

## 3. Results

During the patient collection period, 28,084 patients were admitted to the PACU, of whom 20 remained in the PACU for >2 hours. The general incidence of delayed discharge from PACU was 0.712‰. There were 13 (65%) male patients and 7 (35%) female patients. Sixty percent of the patients were over 65 years of age, and 40% were between 18 and 65 years of age. Most patients had ASA grades II (12, 60%) and III (7, 35%). The mean BMI was (24.45 ± 3.73) kg/m^2^, of which 2 (10%) cases were ≥30 kg/m^2^ and 18 (90%) cases were <30 kg/m^2^ (Table 2).

**Table 2:** Characteristic of patients delayed discharge from PACU.

Characteristics	Frequency (n)	Percentage (%)
Gender		
Female	7	35
Male	13	65
Age (yr)		
≤18	0	0
18~65	8	40
≥65	12	60
ASA grade		
II	12	60
III	7	35
IV	1	5
BMI (kg/m2)		
<30	18 (16)	90
≥30	2 (4)	10

ASA = American society of anesthesiologists, BMI = body mass index

The 3 procedures with the highest proportions were urological surgery (n = 7, 35%), thoracic surgery (n = 4, 20%), and liver surgery (n = 4, 20%). Half of the procedures were performed under general anesthesia, and 9 (45%) procedures were performed under general anesthesia combined with ultrasound-guided peripheral nerve block. The procedure duration was 174 ± 111 minutes; the shortest was 33 minutes, and the longest was 408 minutes. The duration of anesthesia was 239 ± 123 minutes; the shortest was 60 minutes, and the longest was 505 minutes. The length of stay in the PACU was 125 to 285 minutes, with an average of 167 ± 43 minutes (Table 3).

**Table 3:** Surgery and anesthesia information of patients delayed discharge from PACU.

Content	Frequency (n)	Percentage (%)
Type of surgery		
Urologic surgery	7	35
Thoracic surgery	4	20
Liver surgery	4	20
Orthopedic surgery	2	10
Breast surgery	1	5
Combined surgery	1	5
Vascular Surgery	1	5
Type of anesthesia		
General anesthesia	10	50
General anesthesia combined with nerve block	9	45
Monitored anesthesia care	1	5
Procedure duration (minutes, $\bar{x}\pm s$)	174 ± 111	
Anesthesia duration (minutes, $\bar{x}\pm s$)	239 ± 123	
PACU stay duration (minutes, $\bar{x}\pm s$)	167 ± 43	

PACU = post-anesthesia care unit.

Of the 20 patients with delayed discharge from the PACU, 6 (30%) had delayed recovery from anesthesia, and 5 (25%) had surgical complications, including 4 (20%) postoperative bleeding requiring observation or reoperation, 1 (5%) spinal cord hypertension syndrome, 4 (20%) cardiovascular instability, 3 (15%) hypoxia, 1 (5%) inadequate analgesia, and 1 (5%) waiting for operation because the intraoperative pathology was malignant and required reoperation (Table 4). Twelve (60%) patients were transferred to the ward, 4 (20%) patients were transferred to the operating room for reoperation from the PACU, and 4 (20%) patients were transferred to the ICU. There was 1 cardiovascular patient, who developed cardiac arrest died in the ICU, and the other 19 (90%) were discharged uneventfully (Table 5). Details of the 20 patients with delayed discharge from the PACU are shown in Table 6.

**Table 4:** Proportion of factors for delayed discharge from PACU.

Group	Frequency (n)	Percentage (%)
1. Delayed recovery from anesthesia	6	30
2. Surgical complications	5	25
3. Cardiovascular unstable	4	20
4. Hypoxia	3	15
5. Inadequate analgesia	1	5
6. Waiting for operating room	1	5

PACU = post-anesthesia care unit.

**Table 5:** Where patients transferred to from PACU and outcome.

Content	Frequency (n)	Percentage (%)
Where patients transferred to from PACU and outcome		
Operation room	4	20
Ward	12	60
ICU	4	20
Outcome		
Discharged safely	19	95
Significant sequelae	0	0
Death	1	5

ICU = intensive care unit, PACU = post-anesthesia care unit.

**Table 6:** Details of the 20 patients with delayed discharge from the post-anesthesia care unit.

No	Sex	Age (yr)	BMI (kg/m^2^)	ASA	Anesthesia technique	Operation name	Operation duration (min)	Anesthesia duration (min)	PACU duration (min)	Factors	Direction	Outcome
1	M	75	25.35	III	GA	Transurethral resection of bladder tumor	70	145	250	Surgical complications/ postoperative bleeding	Operating room	Discharged safely
2	M	77	23.88	II	GA	Vera laser vaporization + plasma electrotomy + cystoscopy of transurethral prostate	110	180	285	Surgical complications/postoperative bleeding	Operating room	Discharged safely
3	M	75	20.76	IV	GA + NB	Combined thoracoscopic and laparoscopic esophagectomy	408	505	160	Delayed recovery from anesthesia	Ward	Discharged safely
4	M	77	20.76	III	GA	Transurethral plasmotomy of prostate and bladder	110	170	135	Surgical complications/Postoperative bleeding	Ward	Discharged safely
5	M	63	31.14	III	GA	Transurethral resection of bladder tumor	115	160	155	Surgical complications/Postoperative bleeding	Operating room	Discharged safely
6	F	58	31.04	II	GA + NB	Bilateral total knee arthroplasty	120	170	160	Hypoxemia	Ward	Discharged safely
7	F	46	21.64	II	GA	Lumpectomy	33	60	145	Waiting for operating romm for re-operation	Operating room	Discharged safely
8	M	69	28.4	II	GA + NB	Radical resection of cardiac carcinoma	225	310	145	Hypoxia	Ward	Discharged safely
9	M	56	22.49	II	GA	Minimally invasive double-channel endoscopically assisted discectomy + spinal decompression	135	200	145	Cardiovascular unstable/Myeloid hypertension	ICU	Discharged safely
10	M	75	21.2	III	MAC	Right lower limb arterial exploration thrombectomy + arteriography + catheter hemolysis	130	170	140	Delayed recovery from anesthesia	Ward	Discharged safely
11	F	66	16.65	II	GA	Flexible ureteroscopic holmium laser lithotripsy of right pelvis stone	130	230	220	Cardiovascular unstable/arrhythmia	ICU	Discharged safely
12	M	67	29.05	II	GA	Vesicoscopic clot removal	40	85	220	Cardiovascular unstable/Hyperlactemia	ICU	Discharged safely
13	M	75	24.91	III	GA	Transurethral vela laser vaporization enucleation of prostate	100	145	166	Cardiovascular unstable/cardiac arrest	ICU	Death
14	F	69	25.71	II	GA + NB	Thoracoscopic radical resection of right inferior lobe carcinoma + pleural adhesion cauterization + Laparoscopic appendectomy and intestinal adhesiolysis	365	395	130	Delayed recovery from anesthesia	Ward	Discharged safely
15	M	71	24.22	III	GA + NB	Hepatocellular carcinoma radical treatment + cholecystectomy + intestinal adhesion release	360	440	145	Delayed recovery from anesthesia	Ward	Discharged safely
16	M	64	24.22	III	GA + NB	Radical treatment of colon cancer + intestinal adhesion release	165	215	175	Cardiovascular unstable/Hypotension	Ward	Discharged safely
17	F	49	25.85	II	GA + NB	Laparoscopic resection of hepatic hemangioma	130	195	135	Delayed recovery from anesthesia	Ward	Discharged safely
18	F	57	26.04	II	GA + NB	Laparoscopic partial hepatectomy + fenestration and drainage of hepatic cysts	345	445	125	Hypoxia	Ward	Discharged safely
19	M	63	25.65	II	GA	Thoracoscopic segmentectomy	190	255	160	Inadequate analgesia	Ward	Discharged safely
20	F	68	20.03	II	GA + NB	Laparoscopic left hemihepatectomy + cholecystectomy + choledochotomy exploration + T tube drainage	205	295	140	Delayed recovery from anesthesia	Ward	Discharged safely

ASA = American society of anesthesiologists, BMI = body mass index, GA = general anesthesia, ICU = intensive care unit, MAC = monitored anesthesia care, NB = nerve block, PACU = post-anesthesia care unit.

## 4. Discussion

This report provides insight into the characteristics, factors, and outcomes of 20 patients who stayed in the PACU for >2 hours among 28,084 patients admitted to the PACU after surgery in a Chinese class A tertiary comprehensive hospital during the audit period. The percentage of delayed discharge from the PACU was 0.712‰, and the major reasons for delay included delayed recovery from anesthesia, surgical complications, cardiovascular instability, and hypoxia.

There was significant variation in the proportion of delayed discharge from the PACU in different studies, from 0.86% to 61.8%,^[1,6]^ which was significantly higher than that reported in our study. There are several reasons for the large gap between our results and those of the other studies. First, the reasons for delayed PACU discharge include both clinical and nonclinical factors (such as unavailability of beds in the ward, unavailability of a porter for transportation, et al).^[1]^ In some hospitals, nonclinical factors are the main factors.^[1]^ In our hospital, patients who underwent routine surgery were assigned ward beds before surgery and could return directly to their respective beds after surgery if their conditions met the discharge criteria. Therefore, only the clinical factors were included in the present study. Second, there was some variation in the admission and discharge criteria for the PACU in different hospitals. In our hospital, patients admitted to the PACU after removal of the tracheal tube or laryngeal mask airway in the operating room received general anesthesia, which might be a factor in reducing PACU duration compared to removal of the tracheal tube or laryngeal mask airway after surgery in the PACU. Almost all patients admitted to the PACU after surgery were transferred to the clinical area, which might have reduced the PACU stay time compared with those who went home directly. Third, there are differences in medical models and technology levels among countries and hospitals, including staff, clinical care, nursing, and hospital management. This also affects the length of PACU stay. Furthermore, there is no uniform definition of “delayed discharge from the PACU” in current studies.^[7]^ Duration of stay >2 hours, duration >1 hour, or predicting duration according to PACU stay length as a continuous variable was used.^[7]^ These factors should be considered when comparing the study results.

There was a significant proportion of patients aged 65 years or older, with ASA status II and III, general anesthesia, and combined nerve block, staying for more than 2 hours in the PACU, which was consistent with previous surveys.^[8]^ It is likely that patients with ASA class IV or higher were transferred directly from the operating room to the ICU. This suggested that delayed discharge from PACU may occur in patients with relatively few comorbidities and in whom clinicians might not expect the occurrence. Gabriel R.A. reported that morbid obesity (BMI > 40 kg/m^2^) was associated with extended PACU length of stay due to the increased risk for sleep apnea in outpatients surgeries.^[7]^ However, in our survey, only 2 (10%) patients were obese (BMI > 30 kg/m^2^). This discrepancy was likely due to the differences in outpatient and inpatient procedures. More complex procedures (urological surgery [35%], thoracic surgery [20%], and liver surgery [20%]) generally require longer operation and anesthetic durations, special anesthetic techniques, greater impact on the physiological function of patients, higher complication rates, and longer recovery times. Of the 20 patients, the patient with the shortest operative time (33 minutes) underwent lumpectomy. After the surgery, the patient was transferred to the PACU to wait for rapid pathological results. Therefore, the reason for the prolonged PACU length of stay was to wait for the operating room to reoperate because of malignant pathological findings. Procedure-related complications occurred in 5 patients. Four urological procedures required observation in the PACU because of postoperative bleeding, which resulted in delayed discharge. Three patients were transferred back to the operating room to continue the operation and the other patient was transferred directly from the PACU to the ward. One patient who underwent spinal surgery developed a rare spinal hypertension-like syndrome and returned to the ward after treatment in the recovery room. General anesthesia usually requires more anesthetic medication and longer periods of recovery after the procedure than monitored anesthesia care and spinal or epidural anesthesia. However, some studies have reported that dexmedetomidine is associated with a longer recovery time than midazolam/fentanyl combination when used for sedation and analgesia during procedures.^[9]^ In this study, 1 patient who underwent monitored anesthesia care received a continuous intravenous infusion of dexmedetomidine during the procedure and remained in the PACU for 140 minutes before being transferred to the ward.

Several studies have identified that delayed awakening from anesthesia is associated with prolonged stay in the PACU, which is consistent with the current findings.^[7]^ In this study, the majority of prolonged stay in the PACU was due to delayed awakening from anesthesia. The time to awakening from anesthesia was affected by anesthetic and patient factors, the duration of the procedure, and painful stimulation. The main factors responsible for delayed awakening following anesthesia were longer duration of surgery, anesthesia, and high-dose anesthetic medications used during the operative period.^[10,11]^ Despite with the widespread use of rapid acting anesthetic agents, patients often awaken quickly after surgery. Certain underlying organ dysfunction and metabolic disorders may also cause delayed awakening following anesthesia, such as old age, liver disease, renal disease, hypoalbuminemia, electrolyte imbalance, hyper/hypo-glycemia, and hypoxia.^[10,12,13]^ In this study, there were 6 (30%) patients with delayed discharge from the PACU due to delayed awakening from anesthesia in the PACU. The factors included major and longer procedures (three liver surgeries, 2 thoracic surgeries, and 1 vascular surgery), old age (12 [60%] cases ≥ 65 years), use of high-dose opioids and anesthetics, and hypothermia. The use of peripheral nerve blocks significantly reduced PACU length of stay, which may in part be attributed to lower intraoperative opioid requirements.^[14]^ However, in this study, 5 patients who underwent general anesthesia combined with peripheral nerve block developed delayed discharge from the PACU, which may be partly due to the residual effects of anesthetic agents. Moreover, the incidence of delayed awakening due to anesthesia was not high.

In addition to delayed recovery from anesthesia and surgical complications, the most common problems that occurred in the present study were cardiovascular (4 cases, 20%) and hypoxia (3 cases, 15%), among which the worst incidence was cardiac arrest. Patients who involved such issues might experience longer PACU stays than those who did not, which is consistent with other studies.^[8,15,16]^ Hypotension, hypoxia, tachycardia were the critical incidents. Kluger and Bullock also reported that the majority of recovery room incidents were cardiorespiratory related.^[17]^ Rose noted that tachycardia and hypertension in patients were associated with an increased risk of unanticipated ICU admission and mortality.^[18]^ Except for the cardiac arrest case, there were no cases requiring reintubation among 20 patients with delayed discharge from the PACU, which was likely because the patients requiring reintubation were transferred to the ICU unplanned for ventilation, and the length of stay in the PACU was <2 hours.

Among the 20 patients, 12 were transferred to the corresponding ward, 4 were transferred to the operating room for reoperation, and 4 were transferred to the ICU due to cardiovascular instability. Among them, 1 patient who experienced cardiac arrest was transferred to the ICU and died, and the other 19 patients were discharged successfully after treatment. Quinn et al found an association between advanced age, higher ASA grade, and duration of the procedure with unplanned ICU admission after surgery^[19]^ which was similar to the results of our study.

This study has several limitations. First, the retrospective nature of this study is a major limitation. Second, the results of the current study were obtained from a single-center study, and our data did not include neurosurgical procedures, which may have affected the results. We hope to conduct a multicenter prospective survey to explore the risk factors for delayed discharge from the post-anesthetic care unit.

## 5. Conclusions

The incidence of delayed discharge from the PACU was low in this public Class A tertiary comprehensive hospital in China, and it is more likely to occur in the elderly, during major operations, and under general anesthesia. The major reasons for delay were clinical factors, including delayed recovery from anesthesia, surgical complications, cardiovascular instability, and hypoxia. Most patients were safely discharged from the hospital.

## Acknowledgments

We would like to thank our colleagues who helped us with this study. All authors confirm that the results have not been published elsewhere.

## Author contributions

**Conceptualization:** Xiaohui Liu, Xingzhi Cai.

**Data curation:** Xiaohui Liu, Yimin Zhang, Xingzhi Cai.

**Formal analysis:** Xiaohui Liu, Ailan Yu.

**Investigation:** Xiaohui Liu.

**Methodology:** Xiaohui Liu, Huili Kan, Ailan Yu.

**Supervision:** Ailan Yu.

**Writing – original draft:** Xiaohui Liu.

**Writing – review & editing:** Ailan Yu.
